# Foxo1 Promotes Th9 Cell Differentiation and Airway Allergy

**DOI:** 10.1038/s41598-018-19315-z

**Published:** 2018-01-16

**Authors:** Thomas S. Buttrick, Wei Wang, Christina Yung, Kenneth G. Trieu, Kruti Patel, Samia J. Khoury, Xingbin Ai, Wassim Elyaman

**Affiliations:** 1 Ann Romney Center for Neurologic Diseases, Brigham and Women’s Hospital and Harvard Medical School, Boston, MA 02115 USA; 2 Pulmonary and Critical Care, Brigham and Women’s Hospital and Harvard Medical School, Boston, MA 02115 USA; 3 Center for Translational and Computational Neuroimmunology, Columbia University Medical Center, New York, NY 10032 USA; 4 Abu Haidar Neuroscience Institute, American University of Beirut Medical Center, Beirut, Lebanon

## Abstract

T helper 9 (Th9) cells are effector CD4^+^ T cells that are characterized by the production of interleukin-9 (IL-9) and have been associated with allergic responses. Here, we found that the expression of the transcription factor forkhead box O1 (Foxo1) was induced in Th9 and Foxo1 plays a crucial role in the differentiation of Th9 cells. Pharmacological inhibition of Foxo1 or genetic disruption of Foxo1 in CD4^+^ T cells caused a reduction in IL-9 expression while upregulating IL-17A and IFNγ production. Furthermore, chromatin immunoprecipitation (ChIP) followed by luciferase assays revealed direct binding of Foxo1 to both the *Il9* and *Irf4* promoters and induces their transactivation. Lastly, adoptive transfer of Th9 cells into lungs induced asthma-like symptoms that were ameliorated by Foxo1 inhibitor, AS1842856. Together, our findings demonstrate a novel regulator of Th9 cells with a direct implication in allergic inflammation.

## Introduction

Naive CD4^+^ T cells differentiate into one of several functional classes of effector cells upon antigen stimulation. T helper (Th) subsets include the classical Th1 and Th2 lineages and Th17 cells that have been described and extensively characterized^1^. Recently, a new subset of interleukin (IL)-9-producing T helper cells, induced *in vitro* by IL-4 and transforming growth factor (TGF)-β1, has been identified^2,3^. Traditionally associated with the Th2 response, IL-9 is a pleiotropic cytokine that exerts broad effects on a variety of cell types such as mast cells, T cells and epithelial cells^4^. Several transcription factors have been reported to be indispensable for fully differentiated Th9 cells including GATA3^2^, PU.1^5^ and IRF4^6^. Recently we demonstrated that RBP-Jκ and Smad3 cooperate to promote Th9 cell development^7^.

Forkhead box O (FOXO) transcription factors are central to many aspects of cell biology^8^. They translate a variety of environmental stimuli, including insulin, growth factors, nutrients and oxidative stress, into specific gene-expression programs. Foxo1, a member of this family, is involved in T cell homeostasis and survival, and is considered as tumor suppressor in various cell systems^8,9^. Foxo1 has been shown to negatively regulate Th17 cell differentiation and pathogenicity by physically inhibiting the transcription factor RORγt activity, the master regulator of Th17 cells^10^. Moreover, Foxo1 is also involved in the development and function of regulatory CD4^+^ T cells (Tregs) under the control of Akt signaling^11^.

In the present study, we identified Foxo1 as a novel transcription factor required for the differentiation of Th9 cells. We found that Foxo1 expression was induced during Th9 cell polarization *in vitro* and positively regulated the transactivation of *Il-9* and *Irf4*, a process that was reversed by disrupting Foxo1 expression either genetically or pharmacologically. Consequently, inhibition of Foxo1 ameliorated the disease in an asthma-like mouse model.

## Results

### Induced Foxo1 Expression in Th9 Cells

**Figure 1:**
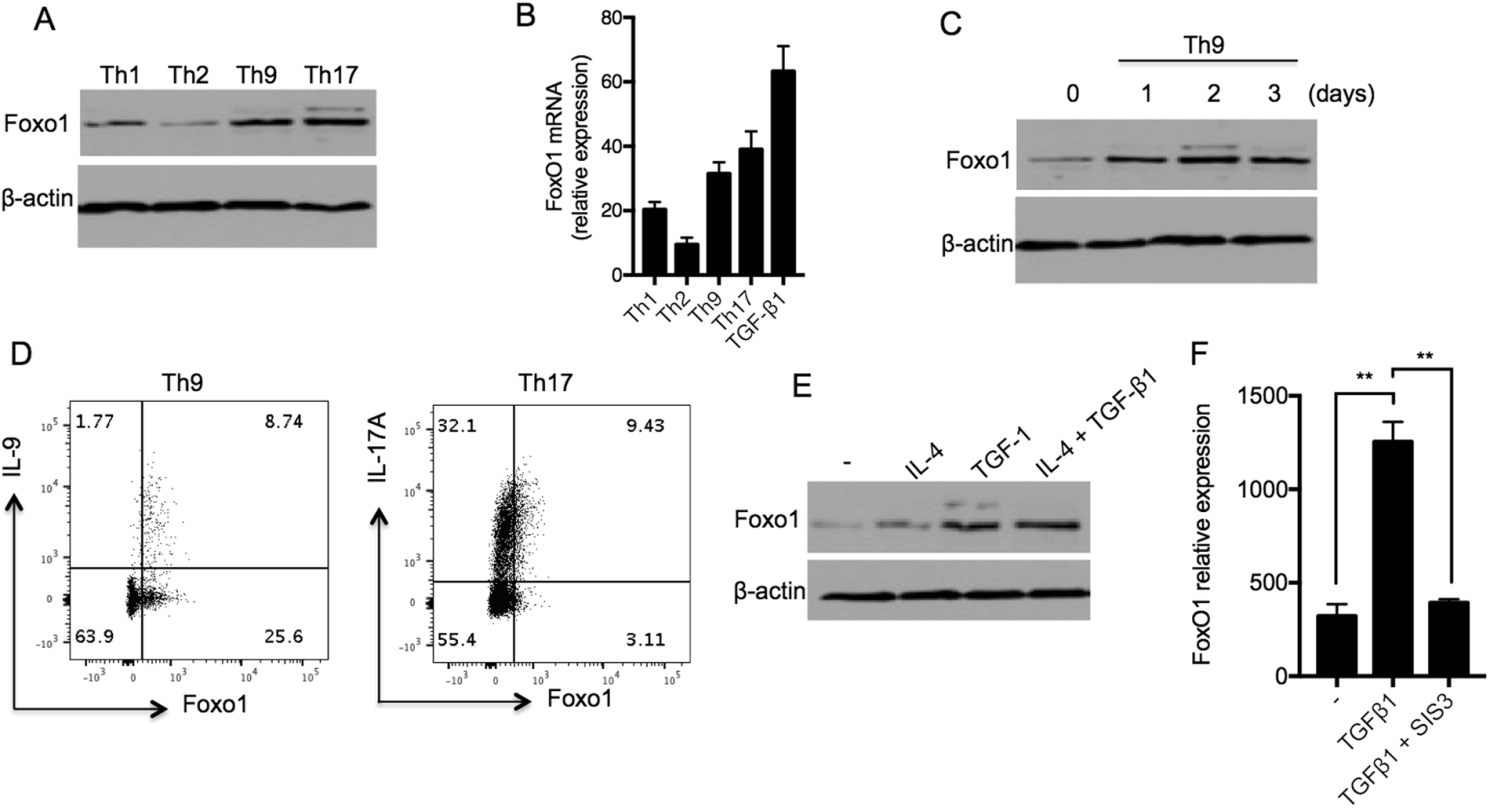
Induced Foxo1 Expression in Th9 Cells. (**A**,**B**) Foxo1 expression comparison in T helper cells. Foxo1 was measured by Immunoblot (**A**) and Taqman PCR (**B**) showing elevated Foxo1 expression in Th9 cells. Naïve CD4^+^ T cells were polarized under Th1, Th2, Th9, Th17 or iTreg (TGF-β1) cell conditions for 4 days and Foxo1 expression was measured by Western blot and Taqman PCR. For the Western blot, β-actin was used as loading control. (**C**) Temporal Foxo1 expression in Th9 cells. Naïve CD4^+^ T cells were polarized under Th9 cell condition for 1–3 days and Foxo1 expression was measured by Western blot. (**D**) Flow cytometry of Th9 and Th17 cells (day 4) analyzed for IL-9 and Foxo1 or IL-17A and Foxo1 expression by intracellular staining. (**E**,**F**) Induced Foxo1 expression in Th9 cells is TGF-β1/Smad3-dependent. (**E**) Naïve CD4^+^ T cells were TCR-activated in the presence of IL-4, TGF-β1 or combined together for 4 days and Foxo1 expression was measured by Western blot. (**F**) Naïve CD4^+^ T cells were differentiated under Th9 cell condition or in the presence of TGF-β1 for 4 days in the presence or absence of Smad3 inhibitor SIS3 (10 μM). Foxo1 expression was measured by Taqman PCR. Data are representative of two independent experiments. **p < 0.01 by Unpaired Student *t* test.

To investigate the role of the transcription factor Foxo1 in Th9 cells, we first measured Foxo1 expression in different T helper subsets including Th1, Th2, Th9 and Th17 cells. Naïve CD4^+^ T cells were polarized *in vitro* under the abovementioned conditions for 4 days and Foxo1 mRNA and protein levels were measured by quantitative Taqman PCR and Western blot, respectively. We found that Foxo1 protein and mRNA were readily expressed by Th9 cells (Fig. 1A,B; Supplemetary Fig. 3A). Controls for T cell polarization were measured by Luminex assay (Supplementary Figure 1). We also measured the temporal Foxo1 expression in Th9 cells polarized for 1–3 days. The time course of Foxo1 protein expression showed that Foxo1 was induced in Th9 cells starting on day 1 after polarization and was maintained on day 3 suggesting that this transcription factor plays a role in the early stages of Th9 cell development and possibly in the maintenance of this lineage (Fig. 1C; Supplementary Fig. 3B). Next, we measured the frequency of IL-9^+^ T cells that co-expressed Foxo1. Using intracellular co-staining of IL-9 and Foxo1 by flow cytometry, we showed that majority of IL-9^+^ CD4^+^ T cells (cells that expressed IL-9 in the Th9 pool) that were polarized for four days, co-expressed Foxo1 (8.74% out of 10.51%) supporting our hypothesis of a potential role of Foxo1 in Th9 cell developments (Fig. 1D).

Given that Th9 cells were differentiated in the presence of the combination of IL-4 and TGF-β1, we measured Foxo1 expression in T cells exposed to either IL-4, TGF-β1, or IL-4 + TGF-β1. We found that while IL-4 treatment had no effect on Foxo1 protein expression compared to control TCR-stimulated T cells, TGF-β1 alone or added to IL-4 induced a noticeable increase in Foxo1 protein level suggesting that TGF-β1 signaling may be involved in the regulation of Foxo1 in Th9 cells (Fig. 1E; Supplementary Fig. 3C). Full-length blots are shown in Supplementary Figure 2. Additionally, quantification of the average of two independent experiments representing Foxo1 protein expression in the T cell conditions outlined in Fig. 1A,C,E, are shown relative to β-actin (Supplementary Figure 3A,B,C).

To test the role of TGF-β1 signaling in Foxo1 expression, we utilized a pharmacological inhibitor of Smad3 activity (SIS3, 10 μM), where Th9 cells were kept untreated or exposed to SIS3 for 4 days. We then measured *Foxo1* mRNA level by quantitative Taqman PCR. Interestingly, we found that inhibition of Smad3 activity prevented the increase in *Foxo1* expression mediated by TGF-β1 treatment confirming the implication of TGF-β1/Smad3 signaling in Foxo1 expression (Fig. 1F).

### Foxo1 Regulates IL-9 Expression in Th9 Cells

**Figure 2:**
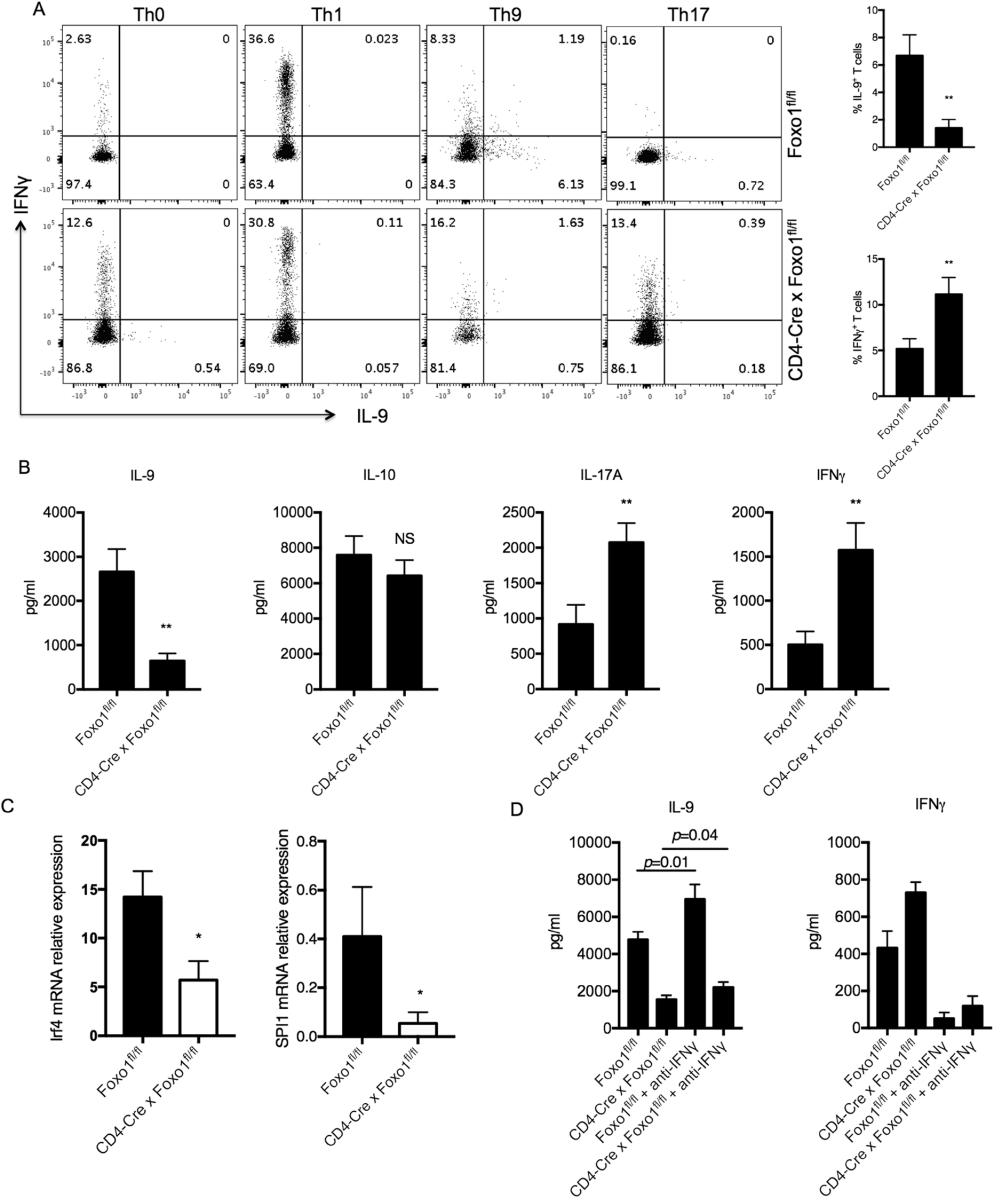
Foxo1 is required for Th9 Cell differentiation. (**A**,**B**) Naïve CD4^+^ T cells purified from splenocytes of conditional Foxo1 knockout mice (*CD4*^*Cre*^*Foxo1*^*fl/fl*^) and littermate controls *(Foxo1*^*fl/fl*^) were differentiated for 4 days under Th0, Th1, Th9 and Th17 cell conditions followed by intracellular cytokine staining (**A**, left panel) and Luminex analysis of cytokine secretion (**B**). Four days following Th9 cell polarization, cells were harvested and stimulated with PMA and ionomycin followed by surface staining for CD4 and intracellular staining for IL-9 (x-axis) and IFNγ (y-axis). Average of three experiments from IL-9- and IFNγ-positive T cells in Foxo1−/− and WT Th9 cells are shown (Fig. 2A, right panel). (**B**) Luminex bead-based cytokine assay of cell-free supernatant collected from Th9 cells 4 days following differentiation. (**C**) Decrease IRF4 and PU.1 expression in Foxo1−/− Th9 cells. IRF4 and PU.1 (*Spi1*) mRNA levels in Foxo1−/− and WT Th9 cells measured by Taqman PCR on day 4 after cell differentiation. (**D**) Effects of IFNγ neutralization on IL-9 expression in Foxo1−/− and WT Th9 cells. Splenic CD4^+^ T cells were isolated from naïve Foxo1−/− and WT mice and were polarized under Th9 cells in the presence or absence of anti-IFNγ neutralizing antibody. IL-9 and IFNγ expression was measured by Luminex assay. Data are representative of three experiments with similar results. **p < 0.01 by Unpaired Student *t* test; NS: not significant.

The transcription factor Foxo1 has been involved in the negative regulation of Th17 cell differentiation^12^ but the role of Foxo1 in Th9 cell development and function has not been described. To study the role of Foxo1 specifically in CD4^+^ T cells polarized under Th9 cell condition, we generated *CD4*^*Cre*^*Foxo1*^*fl/fl*^ mice. Naïve CD4^+^ T cells purified from spleens of conditional Foxo1 knockout mice (Foxo1−/−) or littermate wild-type (WT) controls were differentiated for 4 days under Th9 cell condition followed by intracellular cytokine staining. Control Th0, Th1 and Th17 cell conditions were also generated. We found that genetic deletion of Foxo1 reduced significantly the percentage of IL-9^+^ cells and the level of IL-9 production in Th9 cell conditions compared to WT Th9 cell conditions (~6 fold) by flow cytometry (Fig. 2A, left and right panels) and by Luminex bead-based assay (Fig. 2B). These findings suggest that Foxo1 is a positive regulator of IL-9 expression in Th9 cells. IL-10, another cytokine that is also produced by Th9 cells was not altered in the absence of Foxo1 by Luminex (Fig. 2B), which highlights the specificity of Foxo1 signaling in the regulation of IL-9 transcription under Th9 cell conditions. Interestingly, the decrease in IL-9 expression in Th9 cells lacking Foxo1 was accompanied by an upregulation in IL-17A and IFNγ expression suggesting that Foxo1 may control the plasticity of Th9 cells (Fig. 2A,B). For Th0 and Th17 cell conditions, we detected a striking increase in IFNγ^+^ T cells in these two conditions in the absence of Foxo1 (Fig. 2A), which is in agreement with a previous report showing that Foxo1 is a negative regulator of IFNγ in Th17 cells. However, there was a slight reduction in IFNγ in *CD4*^*Cre*^*Foxo1*^*fl/fl*^ Th1 cells (Fig. 2A). The differential consequence of Foxo1 deletion on IFNγ among T helper subsets may suggest a unique mechanism adopted by Foxo1 in Th1 cells. To further characterize the effects of Foxo1 on Th9 cells, we measured two transcription factors that are required for Th9 cell development, IRF4 and PU.1. We found that Th9 cells lacking Foxo1 exhibited a significant downregulation of *IRF4* and *PU.1* gene expression measured by Taqman PCR 4 days following Th9 polarization (Fig. 2C). To test whether changes in IFNγ levels contributed to the observed Th9 phenotype in the absence of Foxo1, an anti-IFNγ neutralizing antibody was added to WT and Foxo1−/− Th9 cells during differentiation and re-stimulation. We found that although neutralizing IFNγ in Foxo1−/− Th9 cells caused a slight but significant (*p* = 0.04) upregulation in IL-9 expression, it did not reverse the massive downregulation of IL-9 expression in the absence of Foxo1 (Fig. 2D). Thus, IFNγ may play a negative, modulatory role in IL-9 expression.

### Pharmacological Inhibition of Foxo1 Suppresses Th9 Cells

**Figure 3:**
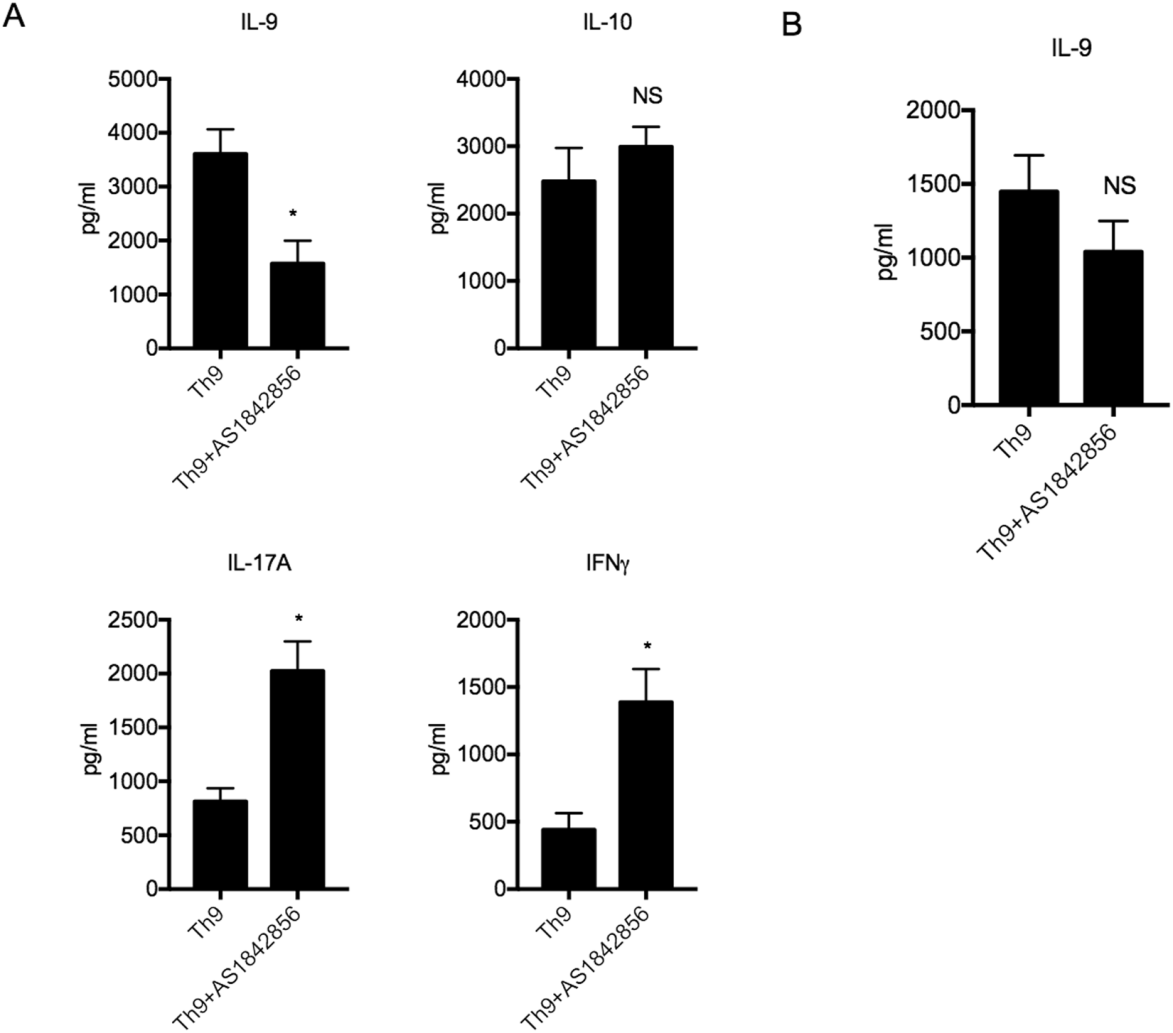
Foxo1 Pharmacological Inhibitor Reduces Th9 Cell Differentiation. **(A**) Naïve CD4^+^ T cells were pretreated with AS1842856 (10 μM) or control vehicle for 2 hours followed by cell polarization under Th9 cell condition for four days. The cytokine profile was analyzed by Luminex assay. (**B**) Th9 cells were differentiated for 4 days followed by the addition of the Foxo1 inhibitor AS1842856 for another round of four days. IL-9 secretion was analyzed by Luminex. Data are representative of three experiments with similar results. *p < 0.05 by Unpaired Student *t* test; NS: not significant.

The Foxo1 inhibitor AS1842856 binds to Foxo1 and disables its ability to transactivate downstream target genes by preventing its interaction with cAMP response element-binding protein^13^. To determine whether pharmacological inhibition of Foxo1 is an effective, therapeutic approach to dampen IL-9 expression, we analyzed the effects of AS1842856 on Th9 cell differentiation *in vitro*. Naïve CD4^+^ T cells were pretreated with AS1842856 or control vehicle for 2 hours followed by cell polarization under Th9 cell condition for four days. At the end of the differentiation, supernatants from these culture conditions were analyzed for cytokine release by Luminex. In agreement with the data collected from Foxo1 deficient mice, we found that targeting Foxo1 pharmacologically was effective in inhibiting IL-9 production by Th9 cells. Moreover, the decrease in IL-9 production correlated with a significant upregulation of IL-17A and IFNγ production while IL-10 level was not changed (Fig. 3A).

Given that both genetic and pharmacological neutralization of Foxo1 inhibits IL-9 production in Th9 cells, we examined whether Foxo1 was required to maintain IL9 production in established Th9 cells. To address this, we exposed Th9 cells 4 days after their differentiation to AS1842856 for four additional days before IL-9 expression was analyzed by Luminex assay. We found that the Foxo1 inhibitor had no significant effect on already differentiated, IL-9 positive cells, suggesting that Foxo1 signaling is mostly required for the early development of Th9 cells (Fig. 3B).

### Foxo1 Binds to *Il9* Promoter and Induces Its Transactivation

**Figure 4:**
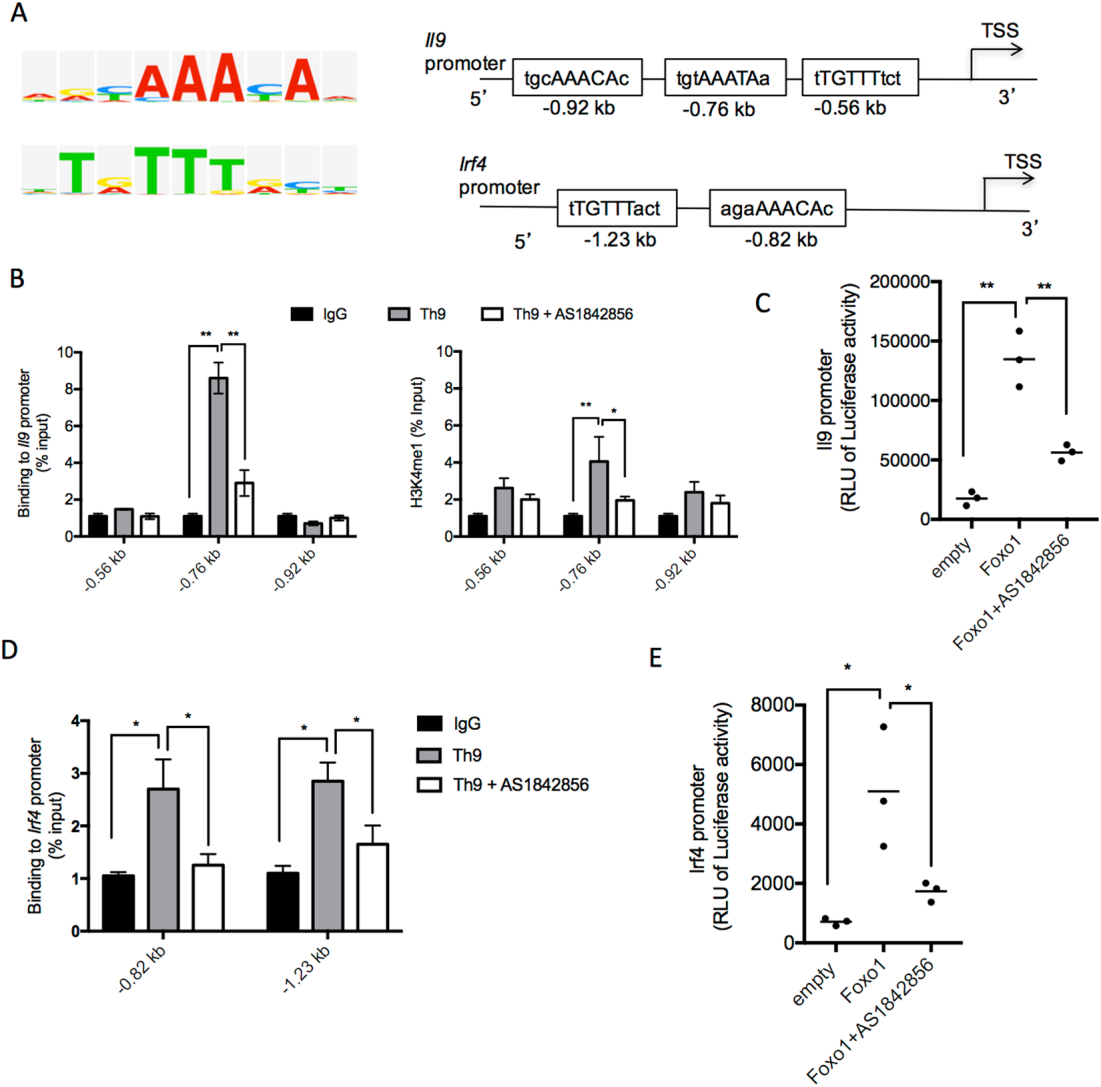
Transcriptional regulation of *Il9* and *Irf4* promoters by FoxO1 in Th9 cells. (**A**) Bioinformatics analysis of the *Il9* and *Irf4* promoters reveals predicted binding sites (boxes) of Foxo1 upstream of the transcription start site (TSS) of these genes. Numbers below diagrams indicate position relative to the TSS (right panel). Consensus binding motifs of Foxo1 are shown (left panel). (**B**) ChIP analysis of Foxo1 binding to the *Il9* promoter in Th9 cells. Naïve CD4^+^ T cells from WT mice were polarized under Th9 cell conditions. ChIP-Sybr Green PCR was performed to determine Foxo1 binding to the *Il9* promoter. Abs used for immunoprecipitation are anti-Foxo1, anti-H3K4me1 and control IgG. Total input DNA before IP was used for normalization of data. The graphs represent quantitative PCR analysis of the ratio of enriched *Il9* promoter with Foxo1 binding sites to the input DNA. Foxo1 binding sites were amplified using *Il9*-specific promoter primers. Data represent mean ± SE of a representative experiment each performed in triplicate. (**C**) *Il9* promoter Luciferase reporter assay. HEK 293 T cells were transfected with Foxo1 together with a constant amount of *Il9* promoter-luciferase vector. Cells were lyzed 48 hrs later and luminescence was measured. (**D**) ChIP analysis of Foxo1 binding to the *Irf4* promoter in Th9 cells. Naïve CD4^+^ T cells from WT mice were polarized under Th9 cell conditions. ChIP-Sybr Green PCR was performed to determine Foxo1 binding to the *Irf4* promoter. (**E**) *Irf4* promoter Luciferase reporter assay. HEK 293 T cells were transfected with Foxo1 together with a constant amount of *Irf4* promoter-luciferase vector. Cells were lysed 48 hrs later and luminescence was measured. Data represent mean ± s.e.m. of a representative experiment each performed in triplicate. *p < *0.01*, **p < 0.005 by Unpaired Student *t* test.

Since Foxo1 has been involved transcriptionally in regulating inflammatory molecules in different immune cells, we hypothesized that Foxo1 may be implicated in the regulation of the *Il9* promoter in Th9 cells. Foxo proteins mainly act as potent transcriptional activators by binding to the conserved consensus motifs TTGTTTAC^14^ and (T/C)(G/A)AAACAA^15^ (Fig. 4A, left panel). Thus, we searched the *Il9* promoter for potential binding sites for Foxo1 using Biobase database. We identified three putative binding sites for Foxo1 at −0.56 kb, −0.76 kb and −0.92 kb upstream of the transcription start site (TSS) of the *Il9* promoter. We also found two potential binding sites upstream of the TSS of the *Irf4* promoter, a key transcription factor required for Th9 cell differentiation (Fig. 4A, right panel). To determine the *Il9* promoter occupancies by Foxo1, the binding motifs were used to design chromatin immunoprecipitation (ChIP) experiments. Primer sets flanking the Foxo1 binding on three sites in the *Il9* were designed to amplify the immunoprecipitated ChIP DNA by qPCR. Naive CD4^+^ T cells were differentiated under Th9 cell polarizing conditions for 24 hours and then analyzed by ChIP-PCR. We detected significant binding of Foxo1 to the −0.76 kb site in the *Il9* promoter in Th9 cells that was associated with an increase in the histone 3 lysine 4 monomethylation (H3K4me1), a mark that characterizes active transcription. As expected, Foxo1 inhibitor abolished Foxo1 recruitment to the *Il9* promoter in Th9 cells and the active transcription mark (Fig. 4B), which correlates with the suppression of IL-9 expression. We confirmed the specificity of Foxo1 binding by the failure to amplify a region of the *Il9* promoter that does not contain Foxo1 binding sites (data not shown). To analyze the functional relevance of the binding of Foxo1 to their target sequence in the *Il9* locus, we investigated the ability of Foxo1 to regulate the activity of the *Il9* promoter in reporter assays. We used reporter construct pGL3-Il9, containing the firefly luciferase gene under the control of the *Il9* promoter. We found that co-transfection of the pGL3-Il9 luciferase reporter construct with a plasmid encoding Foxo1 in 293 T cells resulted in a significant increase in *Il9* transcription that was inhibited by pre-incubating the cells with the Foxo1 inhibitor AS1842856 confirming the specificity of the assay (Fig. 4C).

Further, we investigated the hypothesis that Foxo1 also modulates IRF4 function. Using ChIP assay, we found that Foxo1 is indeed recruited to the −0.82 kb and the −1.23 kb sites upstream of the TSS of the *Irf4* promoter in Th9 cells (Fig. 4D). Next, we measured the functional effect of Foxo1 on the *Irf4* promoter activity. We used a luciferase reporter assay to measure the activity of the *Irf4* promoter in cells overexpressing Foxo1. We found that co-transfection of the pGL3-*Irf4* luciferase reporter construct with a plasmid encoding Foxo1 in 293 T cells resulted in a significant increase in *Irf4* transcription (Fig. 4E). These findings were in agreement with decreased *Irf4* mRNA levels in *CD4*^*Cre*^*Foxo1*^*fl/fl*^ Th9 cells compared to control *Foxo1*^*fl/fl*^ Th9 cells shown in Fig. 2C. Altogether, our findings suggest that Foxo1 exerts a dual regulatory role in Th9 cells by directly binding to and transactivating the *Il9* and *Irf4* promoters.

### Foxo1 Inhibition Is Protective in a Th9 Cell-mediated Asthma

**Figure 5:**
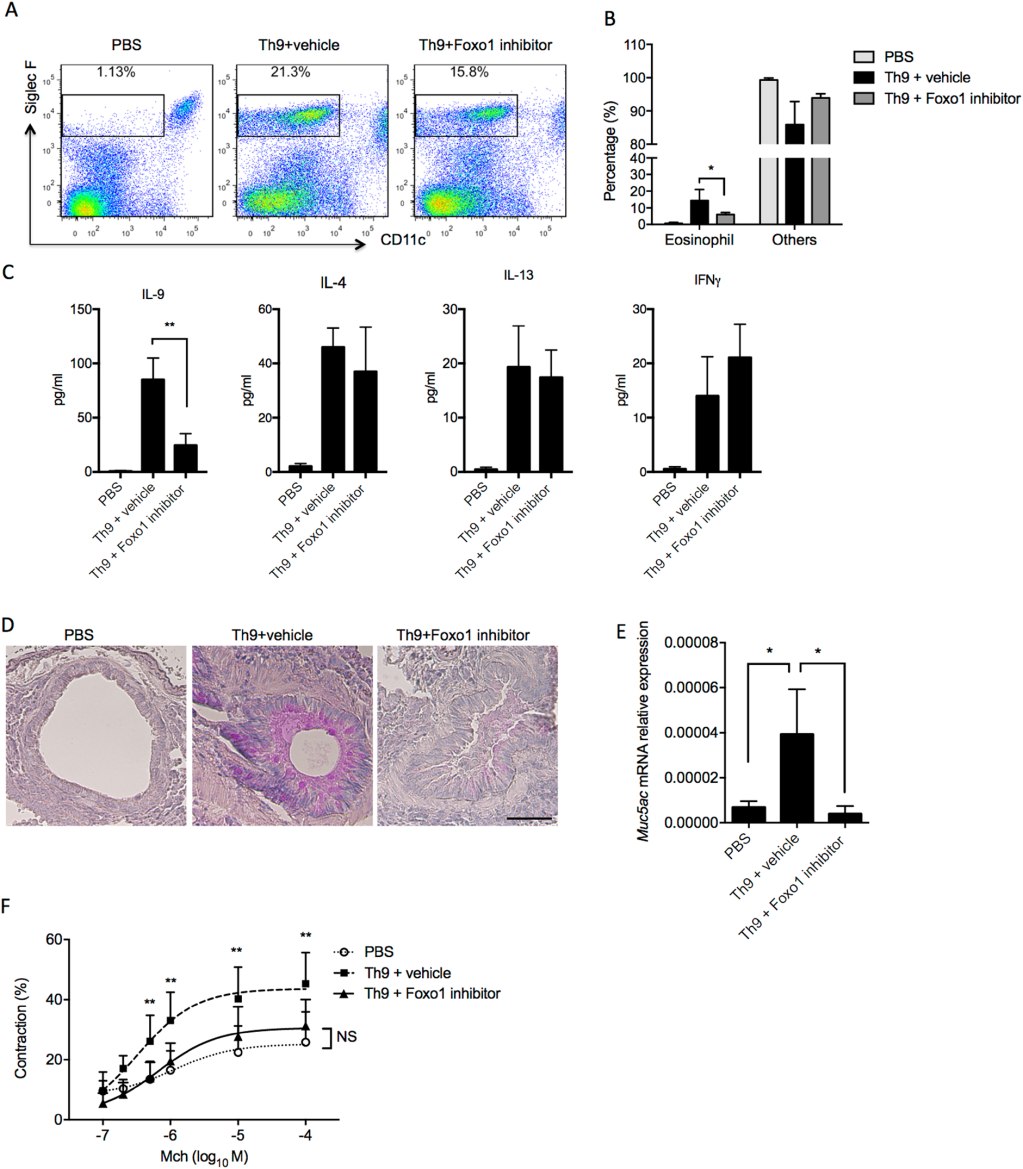
Foxo1 Inhibition Ameliorates Th9 Cell-Induced Asthma. (**A**) Flow cytometry analysis of eosinophils in lungs. Mice received vehicle, Th9 cells and Th9 cells treated with the Foxo1 inhibitor AS1842856 before OVA challenges. Lungs were enzymatically dissociated before staining for CD45 and eosinophil markers, CD11c and Siglec F. The percentages of eosinophils among the CD45^+^ population were shown. Cells from two mouse lungs were pooled in each experiment. Data represent the results in two independent experiments. (**B**) Eosinophil cell counts in BAL of each experimental group. Data showed the percentages of eosinophils and other cell types in BAL of OVA-challenged mice that received vehicle, Th9 cells, and Th9 cells treated with the Foxo1 inhibitor AS1842856. Data represent mean ± s.e.m. from 4 mice of each experimental group. (**C**) Luminex data showing IL-9, IL-4, IL-13, and IFNγ levels in the BAL of mice that received control PBS injection, Th9 cells alone or Th9 cells that were treated with Foxo1 inhibitor. (**D**) Representative PAS staining of mucus in airways from 3 experimental groups. Scale bar, 50 µm. (**E**) *Muc5ac* mRNA levels in each experimental group analyzed by quantitative PCR. Data were normalized to 18 *s*. **(F)** Airway contraction using lung slices from each experimental group. The airway luminal areas at baseline were measured prior to stimulation with increasing doses of methacholine. Contraction of the airway was calculated as the percentage of the reduction in the luminal area following methacholine stimulation. Data represent the mean and SEM from a total of 15 airways, 2 mice in two independent experiments. *p < 0.05. **p < 0.01 by Unpaired Student *t* test. NS, not significant.

Recent studies have shown that Th9 cells have been implicated in airway inflammation and asthma pathogenesis mainly due to the production of IL-9. To examine the role of Foxo1 in the development of inflammatory Th9 cells *in vivo*, we used an adoptive transfer model of ovalbumin (OVA)-specific Th9 cells in which OT-II cells were first polarized *in vitro* under Th9 cell conditions in the presence or absence of Foxo1 inhibitor followed by intra-tracheal (IT) transfer in BALB/C recipients. A third group of mice received PBS treatment only. BALB/C recipients were subjected to OVA nebulization for three consecutive days. On day 4, mice were analyzed for inflammation, mucus overproduction, and changes in airway reactivity. As expected, compared to controls without OT-II cells (PBS only), adoptive transfer of Th9 cells triggered infiltration of eosinophils as analyzed by flow cytometry of lung dissociates stained for cell surface markers SiglecF and CD11c (Fig. 5A). Compared to vehicle-treated Th9 cells, Th9 cells treated with the Foxo1 inhibitor AS184285 reduced the abundance of eosinophils in lungs by ~30% (Fig. 5A). In accordance, the percentages of eosinophils in the bronchoalveolar lavage (BAL) were reduced by half in mice that received AS184285-treated Th9 cells compared to vehicle-treated Th9 cells (Fig. 5B). The decrease in eosinophils by Foxo1 inhibitor AS184285 was associated with a significant reduction in IL-9 but not in IL-4 or IL-13 levels (Fig. 5C). We observed a slight but not significant increase in IFNγ level measured by Luminex assay (Fig. 5C). In addition, vehicle-treated Th9 cells in lungs induced mucus overproduction assayed by PAS staining (Fig. 5D) and by *Muc5ac* gene expression, a molecule that is associated with mucus hypersecretion in the pulmonary tracts (Fig. 5E). In contrast, AS184285-treated Th9 cells showed a dramatic reduction in mucus formation suggesting protective effects (Fig. 5D,E). Consistent with the above inflammatory activity, mice that received vehicle-treated Th9 cells exhibited airway hyper-responsiveness to methacholine measured using prepared, precision cut lung slices compared to PBS controls, while the Foxo1 inhibitor treatment prevented airway responsiveness in the recipient mice (Fig. 5F).

## Conclusion

In the present study, we provide evidence that the transcription factor Foxo1 plays a crucial role in the differentiation of naïve T cells into IL-9-producing T cells. First, Foxo1 is induced in Th9 cells and the majority of IL-9-expressing T cells co-express Foxo1. Second, genetic and pharmacological inhibition of Foxo1 expression causes a dramatic decrease in IL-9 expression. Third, Foxo1 acts transcriptionally on the *Il9* promoter and induces its transactivation. Finally, Foxo1 inhibitor ameliorates asthma-like symptoms induced by adoptive transfer of Ova-specific Th9 cells in mice.

There is heightened interest in understanding the role of Foxo1 in the regulation of adaptive and innate immune systems^16–18^. In primary T cells, Foxo1 is phosphorylated upon TCR ligation, and this process leads to the inhibition of its transcriptional activity by nuclear exclusion^19^. However, Foxo1 posttranslational modification occurs mainly during the early phase of T cell activation to promote cell proliferation clonal cell expansion^20^. In the later stage of TCR engagement, Foxo1 escapes its cytoplasmic retention and translocates to the nucleus to bind to its downstream DNA targets. In the present study, we found that Foxo1 expression is induced during Th9 cell polarization where it binds both *Il9* and *Irf4* promoters and induces their activation. Recently, Foxo1 has been shown to bind the DNA binding domain of RORγt to inhibit its activity in Th17^10^. In agreement with these findings, our study shows that the decrease in IL-9 expression was associated with a noticeable upregulation of IL-17A levels in Th9 cells. The observed increase in IFNγ expression in Th9 cells lacking Foxo1 is in line with previous reports demonstrating that Foxo1 represses T-bet function in memory T cells^21^. Additionally, an inverse correlation between Foxo1 and Tbx21 expression in NK cells was reported and confirmed by Foxo1 genetic disruption leading to an increase in *TBX21* mRNA expression^22^. However, we did not find any alteration in IFNγ production in Th1 cells differentiated according to standard protocol using recombinant IL-12 where T-bet is the master transcription regulator. This suggests that Foxo1 does not regulate directly T-bet function but rather an alternative pathway upstream of T-bet that is not active in Th1 cells. The fact that IFNγ neutralizing antibodies only slightly reverse the decrease in IL-9 expression in Th9 cells deficient in Foxo1 suggests that Foxo1-mediated regulation of IL-9 expression is not exclusively IFNγ-dependent but rather implicates direct and indirect mechanisms.

Our group and others have demonstrated a crucial role of Smad3 signaling in the differentiation of Th9 cells by inducing *Il9* transcription in IRF4-dependent manner^7,23^. Here we found that TGF-β1/Smad3 regulated the expression of Foxo1 in polarized Th9 cells using a pharmacological inhibitor. These findings provide an additional mechanism of action of Smad3 in promoting Th9 cell differentiation that is mediated by Foxo1 signaling.

The role of Foxo1 in the regulating innate immune response in asthma has been recently described. Foxo1 polarizes macrophages into type 2 phenotype by activating IRF4 expression, and inhibition of Foxo1 by AS1842856 attenuates the development of asthmatic lung inflammation^24^. However, to date, Foxo1 was not involved in the regulation of inflammatory T cells in asthma experimental models and particularly Th9 cells. Our present study provides strong evidence demonstrating that Foxo1-IL-9 axis plays a crucial role in the regulation of adaptive immune response in asthma-like mouse models. IRF4 seems to be a common target of Foxo1 in both T cells and macrophages^24^. Together, targeting Foxo1 may have dual impact on the immune responses in asthma by regulating both innate and adaptive immune responses thus ameliorating the disease symptoms. It is intriguing that three recent reports demonstrated a crucial role of Foxo1 in Th9 cell development and function. In one report, the authors investigating the role of mTORC2 in Th9 cells showed that while mTORC2 controls Th9 cell differentiation in a Foxo1/Foxo3a-independent manner, knocking down Foxo1 in CD4^+^ T cells using shRNA strategy reduced their ability to differentiate in Th9 cells^25^, which is in line with our present findings. In a second study investigating the role of IL-7 in Th9-mediated anti-tumor activity, the authors demonstrated that while Foxo1 and Foxp1 play opposite roles in the context of Th9 cell function in tumor regulation, Foxo1 indeed binds to the *Il9* promoter and promotes Th9 cell differentiation and IL-9 production^26^. More recently, and in agreement with our present study, Malik *et al*. provided a detailed analysis of the role of Foxo1 in IL-9 expression in both human and mouse Th9 cells and their pathogenicity in a mouse model of asthma^27^. The authors found that Foxo1 is not only required for IL-9 production in Th9 cells but also in Th17 cells and this process is negatively regulated by AKT signaling.

In summary, our study introduces a novel transcriptional activator of Th9 cells that interacts with well-known regulators of Th9 cells, IRF4 and Smad3. Inhibition of Foxo1 has double impact on both adaptive and innate immune responses in experimental asthma with promising implications in asthmatic patients.

## Materials and Methods

### Mice

*Foxo1*^*fl/fl*^ floxed mice were purchased from Jackson Laboratories and were crossed with *CD4-Cre* mice on the C57BL/6 background for at least 4 generations. Mice were housed in the pathogen-free animal facility at Brigham and Women’s Hospital, in accordance with the guidelines of the Committee of Animal Research at the Harvard Medical School and the National Institutes of Health animal research guidelines as set forth in the Guide for the Care and Use of Laboratory Animals. All studies were performed in compliance with procedures approved by the Harvard Medical School Institutional Animal Care and Use Committee.

### *In vitro* T Cell Differentiation and Cytokine Assay

Naïve CD4^+^ T cells were purified from C57BL/6 WT mice using anti-CD4 beads (Miltenyi, Auburn, CA) and sorted into naive CD4^+^CD44^−^D62L^hi^ T cells by flow cytometry on a FACSAria T cell sorter (BD Biosciences). CD4^+^ T cells were stimulated with plate-bound anti-CD3 (4 μg/ml) (145–2C11; Pharmingen, San Diego, CA) and soluble anti-CD28 (2 μg/ml; Pharmingen) for 3–5 days in a serum-free media (X-VIVO-20; Lonza, Hopkinton, MA) supplemented with 50 μM 2-mercaptoethanol, 1 mM sodium pyruvate, non-essential amino acids, L-glutamine and 100 U/ml penicillin/100 U/ml streptomycin in the presence of recombinant cytokines. Polarization of T cells was in the presence of mouse IL-12 (10 ng/mL) plus anti-IL-4 (10 μg/ml) for Th1, mouse IL-4 (10 ng/mL) plus anti-IFNγ (10 μg/ml) for Th2, human TGF-β1 (3 ng/ml) plus mouse IL-4 (10 ng/ml) for Th9, mouse IL-6 (30 ng/ml) and human TGF-β1 (3 ng/mL) plus anti-IFNγ (10 μg/ml) for Th17. Cells were supplemented with recombinant IL-2 (20 ng/ml) where indicated. All recombinant proteins were from R&D Systems. For intracellular flow cytometry staining, cells were restimulated with 12-O-tetradecanoylphorbol-13-acetate (20 ng/ml; Sigma, St. Louis, MO), ionomycin (300 ng/ml; Sigma), and 2 mM monensin (GolgiStop, BD Biosciences, San Jose, CA) for 4 h at 37 °C. Cells were washed, stained for surface markers, fixed and permeabilized, and anti-IL-9-PE-conjugated antibody was added. For the measurement of cytokines released in the culture supernatants, cell culture supernatants were collected and the secreted cytokines were determined by fluorescent bead-based Luminex technology (Luminex, Austin, TX) for the indicated cytokines, in accordance with the manufacturers’ instructions. AS1842856 was purchased from Calbiochem. Fluorescent conjugated primary mAbs were purchased from BD Pharmingen unless otherwise stated. Neutralizing antibodies were purchased from R&D Systems.

### Expression Analysis by Real-Time PCR

RNA was purified using Stratagene RNA kit and transferred directly into the RT reagent using the Applied Biosystems Taqman reverse transcriptase reagents. Samples were subjected to real-time PCR analysis on an Applied Biosystems PRISM 7000 Sequencer Detection System (Applied Biosystems, Foster City, CA) under standard conditions. Genes analyzed were detected using commercially available assays (Applied Biosystems). For *Foxo1*, assay ID#: Mm00490671_m1; for *Muc5ac*, assay ID# Mm01276718_m1; for *Irf4*, assay ID Mm00516431_m1; for *PU.1*, assay ID Mm03048233_m1. Relative mRNA abundance was normalized against *GAPDH*, assay ID Mm99999915_g1 or 18 *s*, assay ID Mm03928990_g1.

### Western blot

Cells were lysed in RIPA buffer (Thermo Scientific) with a protease inhibitor mixture (Roche Diagnostics) and a phosphatase inhibitor mixture (Sigma-Aldrich); 20 μg total protein was loaded into each well of a SDS-PAGE gel for separation by electrophoresis and then transferred on nitrocellulose membrane. The resulting blots were blocked for 1 h with TBS-Tween 20 containing 5% powder skim milk and then probed for 3 hrs at room temperature with primary rabbit antibody directed against Foxo1 (Cell Signaling). β-actin mouse mAb (Sigma) was used as the loading control. Blots were then washed five times and probed for 1 h with the appropriate HRP-conjugated secondary Ab. Membranes were developed with Immobilon Western Chemiluminescent HRP substrate (Millipore).

### ChIP and qPCR

CD4^+^CD44^−^CD62L^hi^ naive T cells were purified by FACS sorting and were induced toward Th9 and ChIP was performed. Cell lysates were used for immunoprecipitation with anti-Foxo1 antibody (Cell Signaling) and were compared to control IgG. Three regions of the *Il9* promoter and two regions of the *Irf4* promoter containing putative Foxo1 binding sites and were amplified by SYBR Green qPCR (Roche) and quantified in triplicate with the percent of input method. For the *Il9* promoter, the following primers were used: site #1 (−0.56 kb) *Il9* F1: TCTGAGAAGTCGCTCTATGCG and *Il9* R1: TGCTAGCAAGCACAGTTCCA; site #2 (−0.76 kb): *Il9* F2: CCACCCCAGGCACTTTATGT and *Il9* R2: AGTGGCTCAAGGGGTAGAAATG; site #3 (−0.92 kb): *Il9* F3: CCCCTTGAGCCACTGGATAC and *Il9* R3: ACAGAAGTGTGCTGTCTGGT. For the *Irf4* promoter, the following primers were used: site #1 (−82kb): *Irf4* F1: AAGTCAGCGGCAAAAGCTCA a*nd Irf4* R1: AACAGCTAGGCTGACTGAAGG; site #2 (−1.23 kb): *Irf4* F3: GGGGAAAATGGGTGTGACCA and *Irf4* R3: CCATTGTCAGAGCCCTGGTAG.

### Luciferase Reporter Assay

Reporter vector coding for the Firefly Luciferase under the control of the *Il9* promoter encompassing nucleotides −1201 to +52 bp was cloned into the promoterless pGL3 Basic luciferase reporter gene vector (Promega)^28^. *Irf4* promoter encompassing nucleotides −1562 to +122 bp was cloned into pGL3 Basic luciferase reporter gene vector. Reporter assays were carried out as described previously^7^. Briefly, 293 T cells were transfected with 0.4 μg of the reporter vector coding for the Firefly Luciferase under the control of the *Il9* or *Irf4* promoter and with 0.8 μg of the Foxo1 vector. Cells were cultured for 48 hours before harvesting and the relative *Il9* or *Irf4* promoter activity was measured using Promega kit in accordance with the manufacturer’s instructions.

### OVA Model

Cells (0.8 × 10^6^) were delivered intra-tracheally into 8-week-old Balb/c mice (The Jackson Laboratories). After recovery for 24 hours, mice were subjected to aerosolized challenges with 2% ovalbumin (OVA, Sigma) in PBS for 3 consecutive days, 20 minutes each day. Mice were sacrificed on the next day. Blood and lungs were harvested for histological, immunological, and lung slice assays. Two independent experiments were performed. Each group per experiment had 3–4 mice.

### Histology and Flow Cytometry

The right apical lobes were fixed in 4% paraformaldehyde/PBS overnight before paraffin embedding. Tissue sections were collected and analyzed for the level of mucus in airways by PAS (Periodic acid Schiff) staining. For lymphocyte assays, lungs were cut into small pieces and digested for 20 min at 37 °C with collagenase (20 μg/mL, Sigma Aldrich), Dispase II (1 mg/mL, Roche) and DNase I (25 μg/mL, NEB). After enzymatic digestion, tissues were further dissociated by mechanical passage through a wire mesh. Cell pellets were collected by centrifugation. Cells were re-suspended in red blood cell lysis buffer (Sigma Aldrich) for 5 minutes, washed with PBS, stained with antibodies, and analyzed on a BD FACS Aria II flow cytometer. Antibodies included APC-conjugated anti-CD11c (eBioscience), PE-conjugated anti-Siglec-F (BD Bioscience), and PE-Cy7-conjugated anti-CD45 (Biolegend). All antibodies were used at 1:100. Data were analyzed using FlowJo software (Tree Star, Ashland, OR).

### Airway Contraction Assay

Mouse lungs were infused with 1% low-melting agarose (Invitrogen) in HBSS before cutting into slices (200 µm in thickness) using a VF-300 tissue slicer (Precisionary Instruments, San Jose, CA, USA). After overnight recovery in culture, lung slices were transferred into a 12-well plate and stimulated with increasing doses of methacholine (MCh, Sigma-Aldrich). Mid-sized airways with a baseline luminal area between 14,000 and 20,000 µm^2^ were selected for imaging using an inverted microscope (DMI6000B; Leica Microsystems, Buffalo Grove, IL, USA). Each airway was imaged every minute for a total of 5 minutes for each dose of MCh. The airway luminal areas at baseline and after stimulation were measured using NIH Image J software. Contraction of the airway was calculated as the percentage of the reduction in the luminal area.

### Statistical analysis

Data are expressed as mean ± SEM and were compared using Student’s *t* test for experiments with two groups by Prism software v5. Data were considered statistically significant at *P* < 0.05.

## Electronic supplementary material

Supplementary Figures 1-3
