# Supplementary material for: Foxo1 Promotes Th9 Cell Differentiation and Airway Allergy
Source: Sci Rep. 2018 Jan 16;8:818. doi: 10.1038/s41598-018-19315-z (PMC5770389; doi:10.1038/s41598-018-19315-z)
Supplement: Supplementary file 1 — Supplementary Figures 1-3 [file 41598_2018_19315_MOESM1_ESM.pdf]

Foxo1 Promotes Th9 Cell Differentiation and Airway Allergy

Thomas S. Buttrick[1], Wei Wang[2], Christina Yung[3], Kenneth G. Trieu[2], Kruti Patel[1], Samia J. Khoury[1,4], Xingbin Ai[2], and Wassim Elyaman[3]

[1]Ann Romney Center for Neurologic Diseases, Brigham and Women's Hospital and Harvard Medical School, Boston, MA 02115. [2]Pulmonary and Critical Care, Brigham and Women's Hospital and Harvard Medical School, Boston, MA 02115. [3]Center for Translational and Computational Neuroimmunology, Columbia University Medical Center, New York, NY 10032. [4]Abu Haidar Neuroscience Institute, American University of Beirut Medical Center, Beirut, Lebanon.

Address correspondence: Wassim Elyaman, Ph.D.; E-mail: we2152@cumc.columbia.edu, Center for Translational and Computational Neuroimmunology, Columbia University Medical Center, New York, NY 10032, USA. Phone: (212) 305-3609

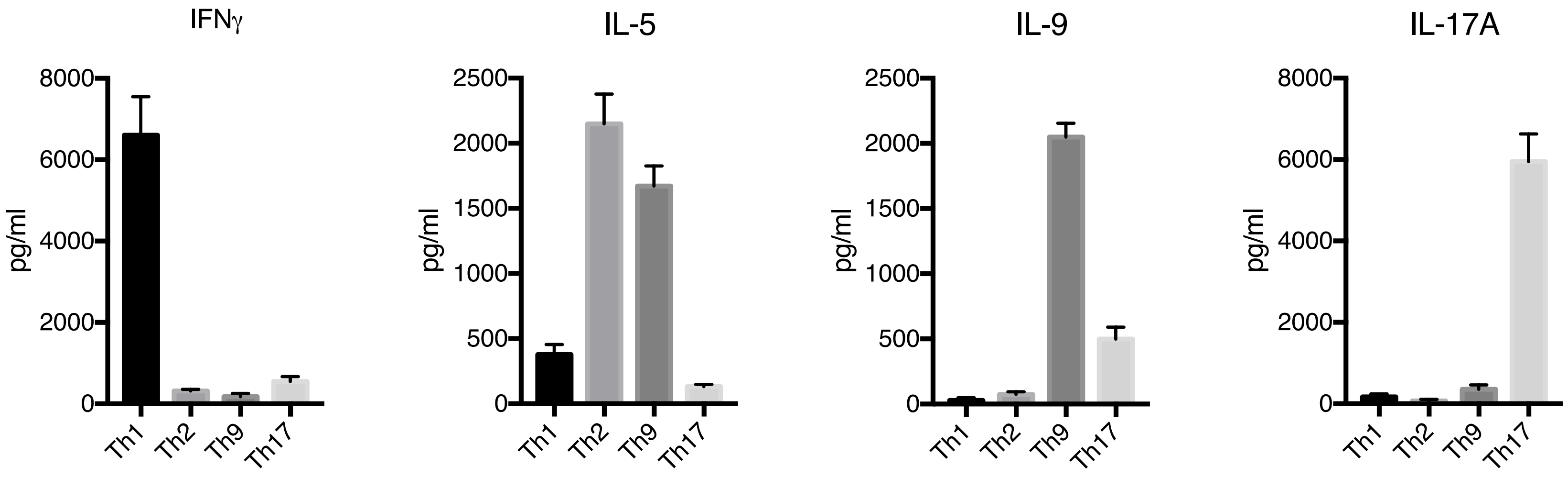

Supplementary Figure 1. Cytokine profile of T helper cells. Naïve $CD4^+$ T cells were differentiated into T helper cells according to the protocol described in the methods section and control cytokines (IFNγ, IL-5, IL-9, and IL-17A for Th1, Th2, Th9 and Th17 cells, respectively) were measured on day 4 after differentiation by bead-based Luminex technology.

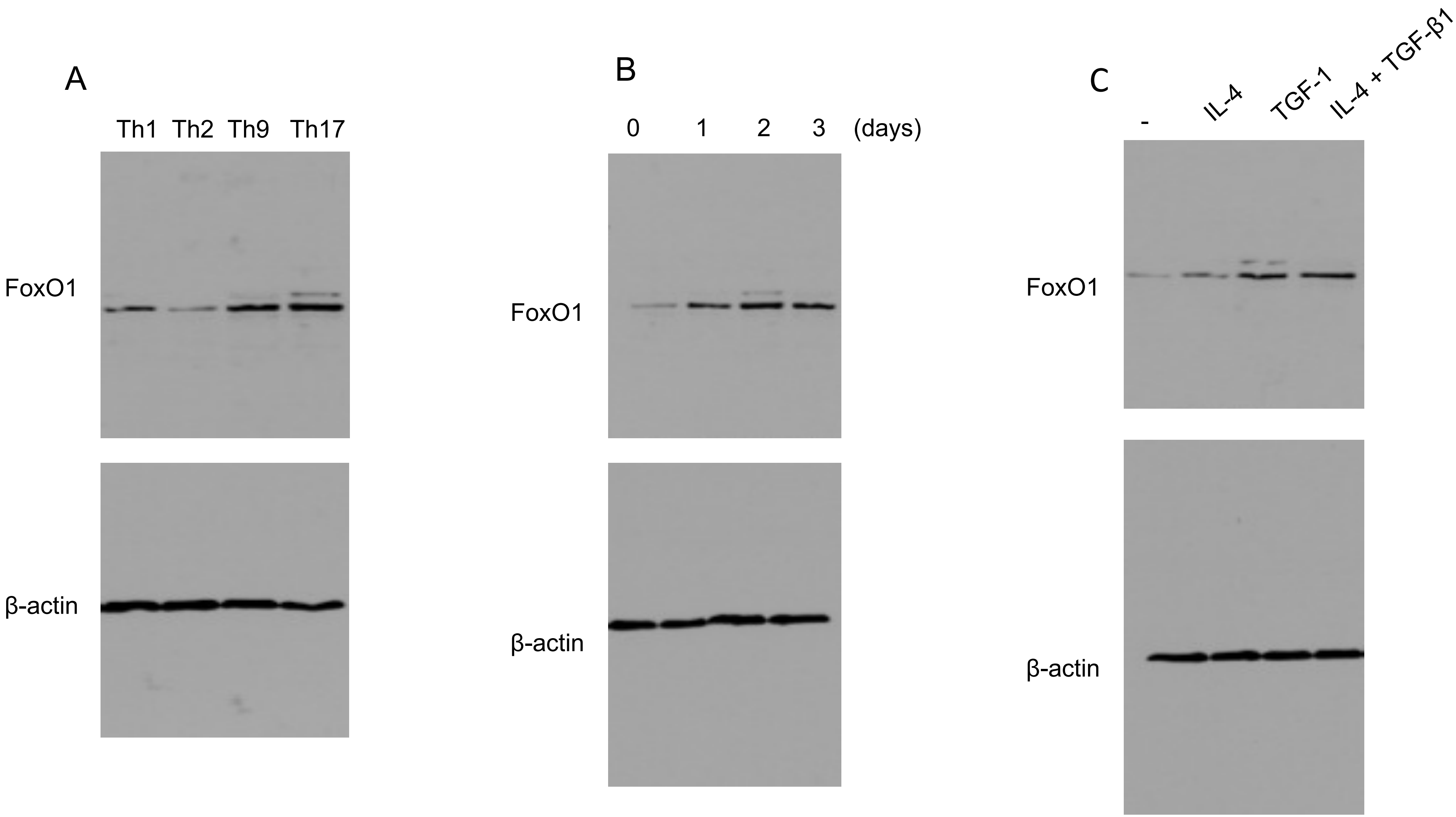

Supplementary Figure 2. Induced Foxo1 Expression in Th9 Cells (Full Western blots corresponding to the data shown in Figure 1A, C, E, respectively).

(**A**) Foxo1 expression comparison in T helper cells. Naïve $CD4^+$ T cells were polarized under Th1, Th2, Th9, or Th17 cell conditions for 4 days and Foxo1 expression was measured by Western blot. (**B**) Temporal Foxo1 expression in Th9 cells differentiated for 0-3 days. (**C**) Induced Foxo1 expression in naïve T cells treated under IL-4 + TGF-β1 (Th9) or with IL-4 or TGF-β1 alone for 4 days. Foxo1 expression was measured by Western blot. β-actin was used as loading control.

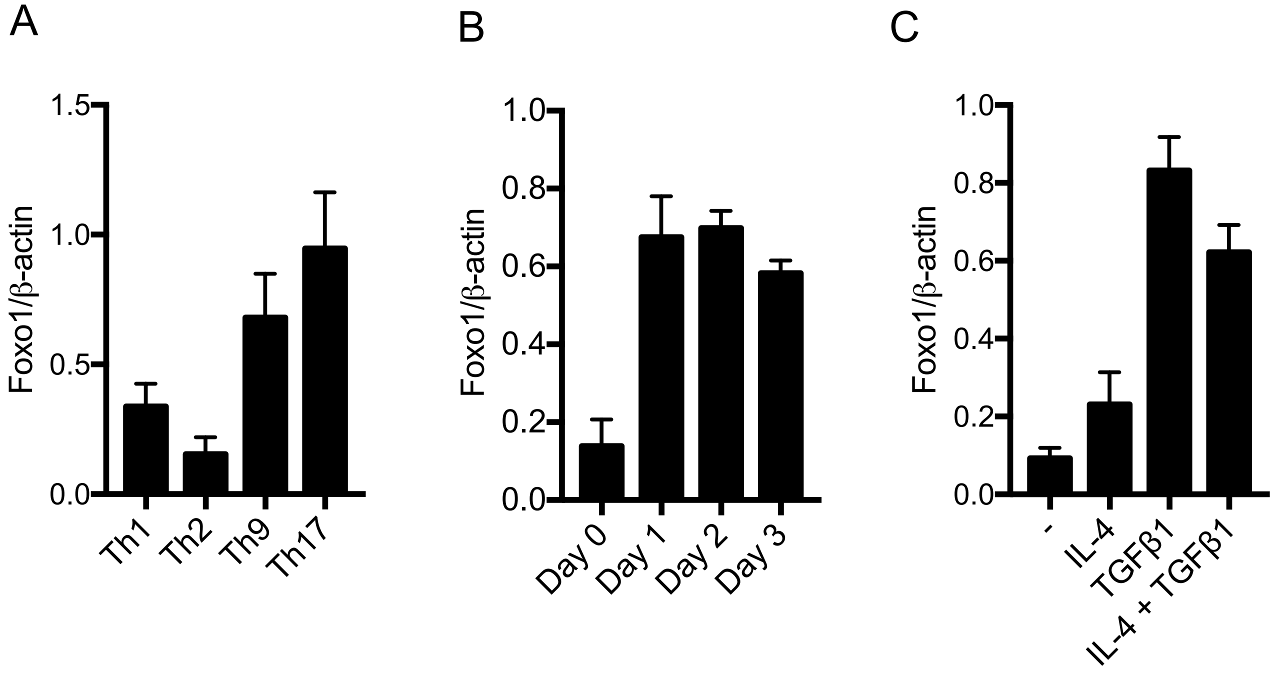

Supplementary Figure 3. Quantification of Foxo1 Protein Expression in T Helper Cells. A, B, C, The intensities of the bands corresponding to the culture conditions described in Figure 1A, C, E, respectively (average of two independent experiments) were calculated using ImageJ software. Data shown are normalized against β-actin levels for each condition.
